# Metabolomic biomarkers of habitual B vitamin intakes unveil novel differentially methylated positions in the human epigenome

**DOI:** 10.1186/s13148-023-01578-7

**Published:** 2023-10-19

**Authors:** Ricardo Costeira, Laila Evangelista, Rory Wilson, Xinyu Yan, Fabian Hellbach, Lucy Sinke, Colette Christiansen, Sergio Villicaña, Olatz M. Masachs, Pei-Chien Tsai, Massimo Mangino, Cristina Menni, Sarah E. Berry, Marian Beekman, Diana van Heemst, P. Eline Slagboom, Bastiaan T. Heijmans, Karsten Suhre, Gabi Kastenmüller, Christian Gieger, Annette Peters, Kerrin S. Small, Jakob Linseisen, Melanie Waldenberger, Jordana T. Bell

**Affiliations:** 1 Department of Twin Research and Genetic Epidemiology, King’s College London, London, SE1 7EH UK; 2 Research Unit Molecular Epidemiology, Institute of Epidemiology, Helmholtz Zentrum München, German Research Center for Environmental Health, 85764 Neuherberg, Germany; 3 Epidemiology, Medical Faculty, University Augsburg, University Hospital Augsburg, 86156 Augsburg, Germany; 4 Molecular Epidemiology, Department of Biomedical Data Sciences, Leiden University Medical Center, 2333 ZC Leiden, The Netherlands; 5 Department of Biomedical Sciences, Chang Gung University, Taoyuan City, Taiwan; 6 Division of Pediatric Infectious Diseases, Department of Pediatrics, Chang Gung Memorial Hospital, Taoyuan City, Taiwan; 7 Department of Nutritional Sciences, King’s College London, London, SE1 9NH UK; 8 Department of Internal Medicine, Section of Gerontology and Geriatrics, Leiden University Medical Center, 2300RC Leiden, The Netherlands; 9 Department of Biophysics and Physiology, Weill Cornell Medicine-Qatar, Doha, Qatar; 10 Institute of Computational Biology, Helmholtz Zentrum München, German Research Center for Environmental Health, 85764 Neuherberg, Germany; 11 German Research Center for Cardiovascular Disease (DZHK), Partner Site Munich Heart Alliance, 80802 Munich, Germany; 12 Institute for Medical Information Processing, Biometry, and Epidemiology (IBE), Ludwig-Maximilians-Universität München, 81377 Munich, Germany

**Keywords:** DNA methylation, Metabolomics, Biomarkers, B vitamins, Folate, Diet

## Abstract

**Background:**

B vitamins such as folate (B9), B6, and B12 are key in one carbon metabolism, which generates methyl donors for DNA methylation. Several studies have linked differential methylation to self-reported intakes of folate and B12, but these estimates can be imprecise, while metabolomic biomarkers can offer an objective assessment of dietary intakes. We explored blood metabolomic biomarkers of folate and vitamins B6 and B12, to carry out epigenome-wide analyses across up to three European cohorts. Associations between self-reported habitual daily B vitamin intakes and 756 metabolites (Metabolon Inc.) were assessed in serum samples from 1064 UK participants from the TwinsUK cohort. The identified B vitamin metabolomic biomarkers were then used in epigenome-wide association tests with fasting blood DNA methylation levels at 430,768 sites from the Infinium HumanMethylation450 BeadChip in blood samples from 2182 European participants from the TwinsUK and KORA cohorts. Candidate signals were explored for metabolite associations with gene expression levels in a subset of the TwinsUK sample (n = 297). Metabolomic biomarker epigenetic associations were also compared with epigenetic associations of self-reported habitual B vitamin intakes in samples from 2294 European participants.

**Results:**

Eighteen metabolites were associated with B vitamin intakes after correction for multiple testing (Bonferroni-adj. *p* < 0.05), of which 7 metabolites were available in both cohorts and tested for epigenome-wide association. Three metabolites — pipecolate (metabolomic biomarker of B6 and folate intakes), pyridoxate (marker of B6 and folate) and docosahexaenoate (DHA, marker of B6) — were associated with 10, 3 and 1 differentially methylated positions (DMPs), respectively. The strongest association was observed between DHA and DMP cg03440556 in the *SCD* gene (effect = 0.093 ± 0.016, *p* = 4.07E−09). Pyridoxate, a catabolic product of vitamin B6, was inversely associated with CpG methylation near the *SLC1A5* gene promoter region (cg02711608 and cg22304262) and with *SLC7A11* (cg06690548), but not with corresponding changes in gene expression levels. The self-reported intake of folate and vitamin B6 had consistent but non-significant associations with the epigenetic signals.

**Conclusion:**

Metabolomic biomarkers are a valuable approach to investigate the effects of dietary B vitamin intake on the human epigenome.

**Supplementary Information:**

The online version contains supplementary material available at 10.1186/s13148-023-01578-7.

## Background

DNA methylation (DNAm) is an important epigenetic mechanism in development and over the lifecourse. In mammals, DNAm typically occurs through the transfer of methyl groups from S-adenosylmethionine (SAM) to cytosine residues at CpG dinucleotides. SAM is the product of one carbon metabolism, which includes the folate (B9) and methionine cycles and utilizes nutrients as substrates [1, 2]. SAM and DNAm levels are influenced by diet and the intake of nutrients [2], particularly B vitamins. Vitamins B6 and B12 are cofactors involved in the regulation of the catalytic activity of enzymes from the folate cycle where folate is the main substrate [3]. DNA methyltransferases (DNMTs) convert SAM into S-Adenosylhomocysteine (SAH), a metabolic precursor of homocysteine (hcy), and folate and vitamins B6 and B12 in the diet can reduce serum homocysteine (hcy-s) levels and promote its re-methylation to methionine [4]. In contrast, hcy accumulation and hyper-homocysteinemia can arise from nutritional deficiencies of B vitamins and lead to DNMT inhibition and DNA hypomethylation [5, 6].

Recently, two large-scale studies explored evidence for epigenome-wide association between self-reported B vitamin intakes and blood-based DNA methylation profiles. Chamberlain et al. [7] explored differential methylation with dietary intakes estimated from food frequency questionnaires (FFQs) in 5186 adult participants from the Melbourne Collaborative Cohort Study, reporting one association with B2 intake. Mandaviya et al*.* [8] also explored methylation associations with B vitamin intakes estimated from FFQs in a meta-analysis of 5841 participants across 10 European and North American cohorts, identifying multiple differentially methylated positions (DMPs) and regions associated with folate and B12 intakes. All but one signal showed an inverse correlation between folate intakes and whole blood DNAm levels. Overall, there is relatively modest overlap across the results from the two studies in adults [9], which may in part be attributed to differences in study design and methodology, or weak association between dietary intake of B vitamins and DNA methylation. A further study by Joubert et al. [10] reported associations between maternal intake of B vitamins in pregnancy and blood based methylation in newborns, but these results are not replicated in samples from adult participants.

FFQs are commonly used to assess habitual nutrient intakes in epidemiological studies due to their practicality for regular assessment of diets over time [11], but they also have limitations. By design, FFQs include a finite list of food items and portion sizes, and have limited specificity on food preparation and types of food [12]. Moreover, food intake greatly depends on ethnic, social, and cultural background and FFQs need to be well-tailored to the study population [11]. FFQs also suffer from social desirability bias where participants omit specific foods and beverages, therefore misreporting can occur. Further imprecision in the estimation of nutrient intakes from FFQ derived food intake estimates originate from the application of food composition databases [12].

Inaccurate dietary assessments may limit our understanding of the impact of B vitamins on DNAm. In contrast, biochemical markers may provide more accurate measures of specific aspects of dietary intake for the time point of biospecimen collection [13]. Metabolomics—the global assessment of all metabolites present in a biological sample—has major value for biomarker discovery in nutrition. Multiple cohort and intervention studies have identified metabolomic biomarkers of dietary patterns, foods and beverages such as tea, coffee, wine, cocoa, citrus fruits, fish, red meat, whole-grain products and more [14, 15]. Recently, Posma et al. [16] showed that urinary metabotypes collected three weeks apart are more stable than 24h dietary recalls, and that up to 67 nutrients, including folate and vitamin B6, can influence the urinary metabotype of participants.

In this study we aimed to use blood metabolomic biomarkers to investigate the effects of dietary B vitamins on blood DNAm variation. We first identified blood metabolomic biomarkers of folate and vitamins B6 and B12 dietary intakes in population-based samples from the UK, and subsequently explored their metabolomic-epigenetic associations in European cohorts. The metabolomic-epigenetic associations were compared with epigenetic associations of self-reported dietary B vitamin estimates in the current study and from previous work.

## Results

**Fig. 1:**
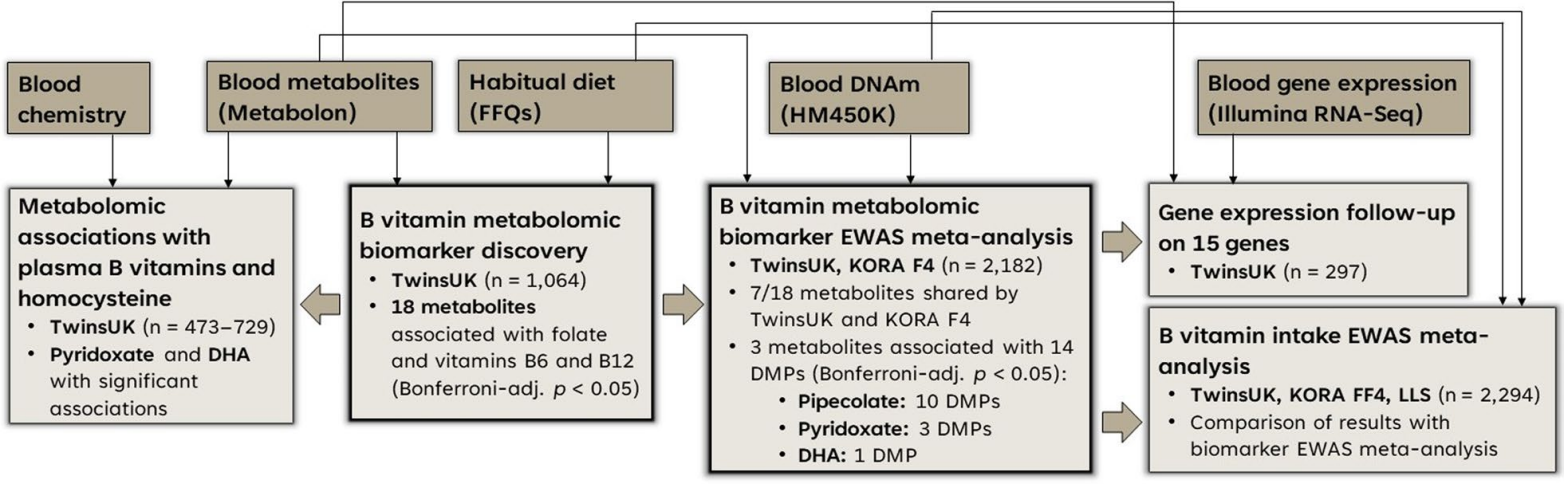
Data analysis workflow and main results from this study

Serum metabolites related to the intake of B vitamins were identified for use in downstream epigenetic association analyses aiming to detect differentially methylated signals related to B vitamin intakes in > 2000 participants of European ancestry (Fig. 1).

### B vitamin metabolomic biomarker discovery

Discovery of blood metabolomic biomarkers related to the intake of folate and vitamins B6 and B12 was conducted in 1064 samples from the TwinsUK cohort (Additional file 1: Table S1). Thirty-one metabolites were associated with the intake of one or more B vitamins (Bonferroni-adjusted *p* < 0.05; Additional file 1: Table S2). Of these, 18 metabolites were annotated to a known biochemical compound and were explored in downstream analyses.

**Fig. 2:**
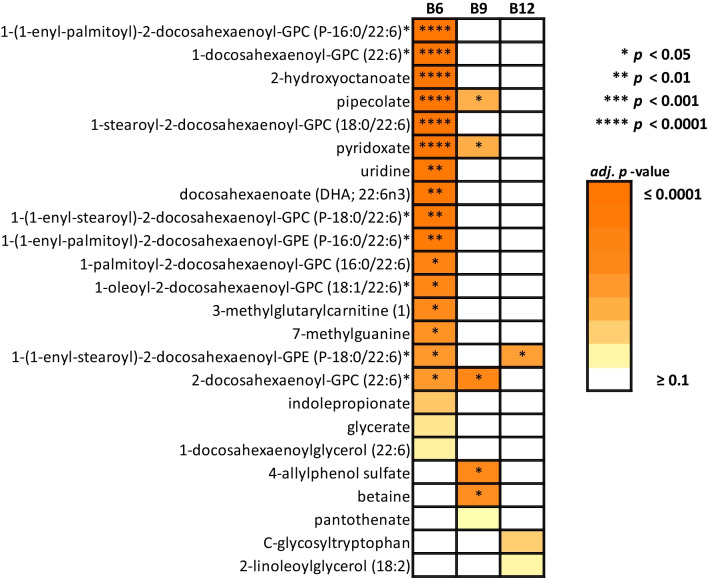
Peak metabolomic associations for folate (B9) and vitamins B6 and B12 habitual intakes in the Metabolon platform (Bonferroni-adj. *p* < 0.1)

Among the 18 metabolites identified, 16, 5 and 1 metabolites were associated with the intakes of folate and vitamins B6 and B12, respectively (Bonferroni-adj. *p* < 0.05; Fig. 2). Compounds 2-docosahexaenoyl-GPC (22:6)*, pyridoxate and pipecolate were associated with both folate and vitamin B6, and 1-(1-enyl-stearoyl)-2-docosahexaenoyl-GPE (P-18:0/22:6)* was associated with both vitamins B6 and B12. Other compounds were associated with one B vitamin alone after multiple testing correction. Pyridoxate and pipecolate levels were not correlated with the levels of other biomarkers identified and fatty acid molecules had varying degrees of direct correlation with each other (Additional file 2: Figure S1).

The strongest metabolomic associations were observed for vitamin B6 with 1-(1-enyl-palmitoyl)-2-docosahexaenoyl-GPC (P-16:0/22:6)* (*b* = 0.335 ± 0.058, adj. *p* = 5.09E−06, R^2^_c_ = 0.591), followed by 1-docosahexaenoyl-GPC (22:6)* (*b* = 0.317 ± 0.058, adj. *p* = 4.88E−05, R^2^_c_ = 0.480), 2-hydroxyoctanoate (*b* = − 0.313 ± 0.060, adj. *p* = 1.55E−04, R^2^_c_ = 0.451) and pipecolate (*b* = 0.296 ± 0.059, adj. *p* = 4.76E−04, R^2^_c_ = 0.489). The strongest association identified for folate was with 2-docosahexaenoyl-GPC (22:6)* (*b* = 0.001 ± 0.0003, adj. *p* = 0.012, R^2^_c_ = 0.400), and the only compound associated with B12 was 1-(1-enyl-stearoyl)-2-docosahexaenoyl-GPE (P-18:0/22:6)* (*b* = 0.055 ± 0.013, adj. *p* = 0.030, R^2^_c_ = 0.599). Docosahexaenoate (DHA; 22:6n3) was associated with vitamin B6 (*b* = 0.268 ± 0.057, adj. *p* = 2.53E-03, R^2^_c_ = 0.519).

Of the 18 metabolites identified in our main analysis, 11 were annotated to the lipid superpathway with 5 of them playing a role in phospholipid metabolism and two belonging to the plasmalogen or lysolipid subpathways (Additional file 2: Figure S2). Other metabolic pathways found in our results included nucleotide and amino acid pathways. Pyridoxate — associated with vitamin B6 (*b* = 0.290 ± 0.059, adj. *p* = 8.34E-04, R^2^_c_ = 0.407) and to a lesser extent with folate (*b* = 0.0013 ± 0.0003, adj. *p* = 0.039, R^2^_c_ = 0.388) intakes — was annotated to the vitamin B6 metabolism pathway.

#### Sensitivity analyses

Three sensitivity analyses were carried out to assess the specificity of the 18 blood metabolic biomarkers of B vitamin intakes. First, we explored whether total energy intake and overall diet quality, estimated using the ‘AHEI-2010’ diet score [17], affected the biomarker results.

All 18 metabolomic associations reported in the main analysis remained nominally significant after adjusting for diet quality and energy intake (*p* < 0.05) (Additional file 1: Table S3), and 10 metabolomic associations remained significant after multiple testing correction, including 1-docosahexaenoyl-GPC (22:6)*, pyridoxate, uridine and DHA metabolites later on used in the downstream epigenome-wide association analysis. New associations were also identified in the sensitivity analysis, including folate intake associated with theanine and vitamin B12 associated with five other metabolites (Additional file 1: Table S3).

Second, we assessed the specificity of the 18 metabolite biomarkers of B vitamin intakes, by testing their association with the intake of 38 other nutrients estimated from FFQs (see Methods). Metabolomic associations with other nutrients were identified (Additional file 1: Table S4), and all B vitamin metabolomic biomarkers that were assessed in the downstream epigenome-wide association analysis below (1-docosahexaenoyl-GPC (22:6)*, 7-methylguanine, betaine, pipecolate, pyridoxate, uridine and DHA) were associated other nutrients. Pipecolate and DHA had the largest number of associations reported in this analysis with 13 and 16 other nutrient associations found for each metabolite at a Bonferroni-adj. *p* < 0.05 threshold extrapolated from the full 756 metabolite panel (Additional file 1: Table S5).

The final sensitivity analysis explored if the identified B vitamin intake biomarkers could be validated by assessing their association with the levels of folate, vitamin B12 and hcy in plasma, and hcy in serum (hcy-s), which were available in sample subsets for 473–729 individuals in the TwinsUK cohort (Additional file 1: Table S1). Pyridoxate and betaine were associated with levels of folate after multiple testing correction (Bonferroni-adj. *p* < 0.05) and 1-(1-enyl-stearoyl)-2-docosahexaenoyl-GPE (P-18:0/22:6)* was associated with levels of vitamin B12 nominally (*p* < 0.05; Additional file 1: Table S6). Directions of effect for circulating folate and vitamin B12 matched the directions of effect of the main analysis (Additional file 1: Table S2 and S6).

Of the 18 metabolomic B vitamin biomarkers, 10 were nominally associated with either hcy, hcy-s, or both (*p* < 0.05; Additional file 1: Table S6). Compounds 1-docosahexaenoyl-GPC (22:6)*, 7-methylguanine, pyridoxate and DHA, used in the downstream epigenome-wide association analysis, were associated with both. 7-methylguanine and DHA had the strongest associations with hcy and/or hcy-s in this sensitivity analysis (Bonferroni-adj. *p* < 0.05). As expected, all significant associations between homocysteine and blood metabolites showed the opposite direction of association effect to that observed between the intake of B vitamin and their respective blood metabolite biomarker (e.g., DHA levels increase with B12 intake and hcy levels lower with increased DHA in blood; Additional file 1: Tables S1 and S6). This result was expected, because folate and vitamins B6 and B12 break down homocysteine to methionine.

**Table 1:** B vitamin metabolomic biomarker results from the TwinsUK and KORA F4 epigenetic association meta-analysis (Bonferroni-adj. *p* < 0.05, HetISq < 75 and HetPVal ≥ 0.05; n = 2182)

DMP	UCSC gene(s) (± 10 kb)	Compound name	Effect size	SE	*p* value	Direction*	HetISq	HetPVal
cg20732160 ( ~)	*PFKFB4/UCN2*	Pipecolate	− 2.91E−02	4.32 E−03	1.68 E−11	– –	0.00	0.945
cg10589813 (@)	*CEBPB*	Pipecolate	− 2.93 E−02	4.43 E−03	3.49 E−11	– –	0.00	0.825
cg18120259 ( ~)	*LOC100132354*	Pipecolate	− 2.67 E−02	4.36 E−03	9.18 E−10	– –	0.00	0.510
cg11800635 ( ~)	*LOXL3/DOK1/M1AP*	Pipecolate	− 3.26 E−02	5.61 E−03	6.35 E−09	– –	0.00	0.385
cg08616943 (@)	*LOC646329/MIR29A/* *MIR29B1*	Pipecolate	− 2.18 E−02	3.76 E−03	6.94 E−09	– –	0.00	0.942
cg03523740 (^)	*KPNA6/TXLNA*	Pipecolate	− 1.69 E−02	2.97 E−03	1.14 E−08	– –	0.00	0.708
cg12054453 ( ~)	*VMP1/MIR21/* *DM119512/U6*	Pipecolate	− 6.60 E−02	1.18 E−02	2.26 E−08	– –	46.60	0.171
cg26841068 ($)	*PRELP/OPTC*	Pipecolate	− 2.26 E−02	4.17 E−03	5.66 E−08	– –	0.00	0.587
cg13442969 (~ , #)	*DYRK2*	Pipecolate	− 2.87 E−02	5.34 E−03	7.84 E−08	– –	57.50	0.125
cg27180443 (^)	*SCARB1*	Pipecolate	− 1.72 E−02	3.23 E−03	1.05 E−07	– –	0.00	0.570
cg02711608 (~ , #, %)	*SLC1A5*	Pyridoxate	− 3.76 E−02	6.23 E−03	1.63 E−09	– –	2.30	0.312
cg06690548 ( ~)	*SLC7A11*	Pyridoxate	− 7.33 E−02	1.30 E−02	1.65 E−08	– –	71.50	0.061
cg22304262 (~ , #)	*SLC1A5*	Pyridoxate	− 4.10 E−02	7.64 E−03	8.27 E−08	– –	0.00	0.444
cg03440556 ( ~)	*SCD*	DHA	9.25 E−02	1.57 E−02	4.07 E−09	+ +	0.00	0.366

Location: ~ gene body, ^ TSS1500, $ 3’UTR, # 5’UTR, % 1st exon, @ intergenic region

*Directions of effect in TwinsUK and KORA, respectively

Extending these results to a metabolome-wide analysis, we observed 14, 15, 23 and 47 metabolites associated with the circulating levels of folate, vitamin B12, hcy and hcy-s, respectively (Bonferroni-adj. *p* < 0.05; Additional file 1: Table S7). Here, strongest signals were identified between vitamin B12 and pantothenate (vitamin B5), and homocysteine (hcy/hcy-s) and pseudouridine. The strongest signal for folate remained pyridoxate metabolome-wide (*b* = 0.050 ± 0.006, adj. *p* = 7.36E−13).

### B vitamin metabolomic biomarker epigenome-wide meta-analysis

The 18 metabolomic biomarkers identified for folate and vitamins B6 and B12 intakes were explored for associations with blood DNAm levels at 430,768 autosomal probes in the TwinsUK and KORA F4 cohorts (n = 2182; Additional file 1: Table S8). The KORA F4 dataset included 7 of the 18 metabolites under investigation, therefore subsequent analyses focused on the 7 common metabolomic biomarkers of B vitamin intakes: 1-docosahexaenoyl-GPC (22:6)*, 7-methylguanine, betaine, pipecolate, pyridoxate, uridine and DHA. Epigenetic analyses were carried out within each cohort, taking into account cohort-specific confounders, and results were meta-analysed.

**Fig. 3:**
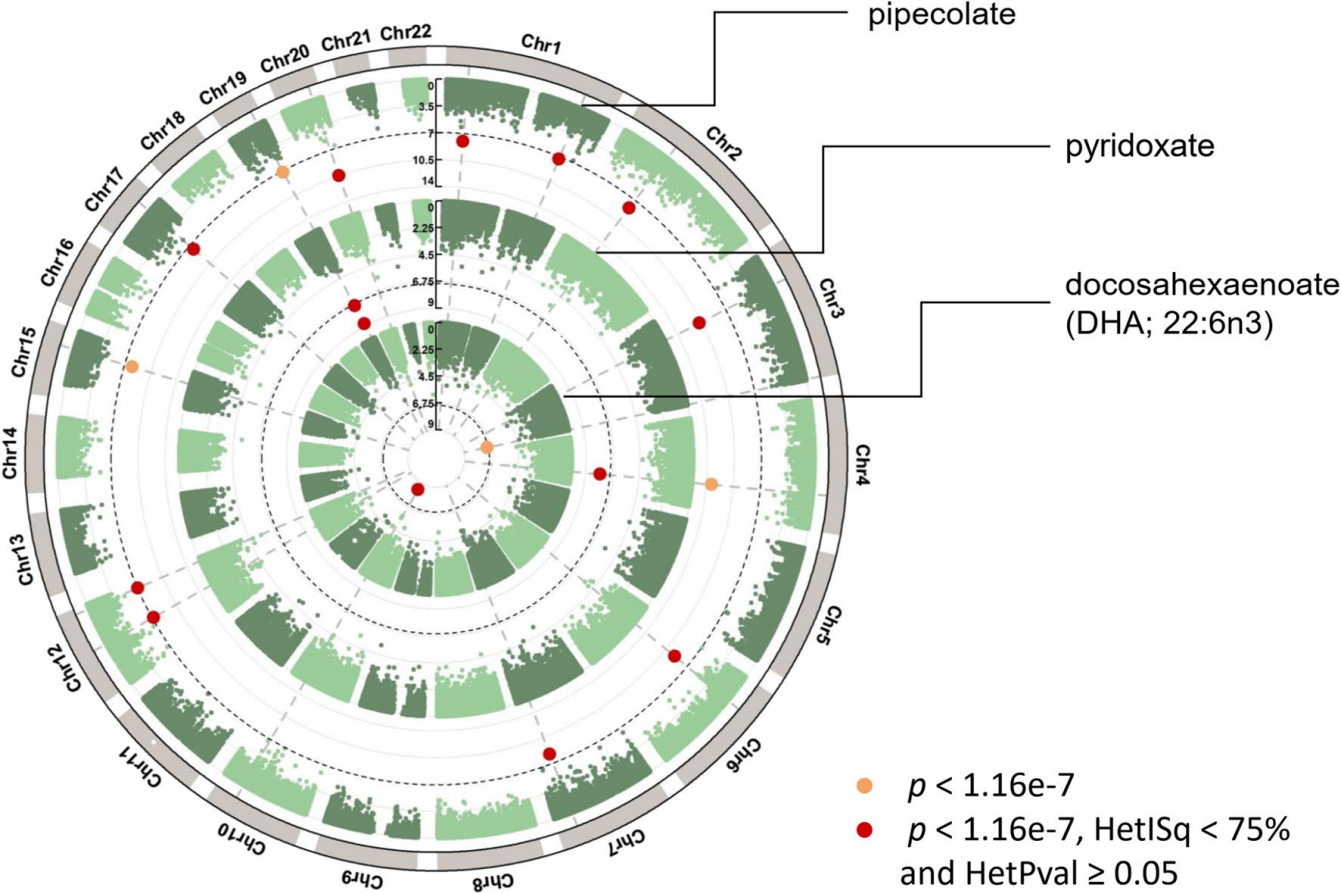
Circular Manhattan plot of the B vitamin metabolomic biomarker results from the TwinsUK and KORA F4 epigenetic association meta-analysis (n = 2182)

Three of the 7 metabolites in the epigenome-wide association meta-analysis showed significant differential DNAm levels in whole blood (Fig. 3). Pipecolate, pyridoxate and DHA were associated with 10, 3 and 1 DMPs each, respectively (Bonferroni-adj. *p* < 0.05, HetISq < 75% and HetPval ≥ 0.05). Pipecolate and pyridoxate are potential biomarkers of vitamin B6 and folate, while DHA is a potential metabolite biomarker of vitamin B6 intake alone, although they also show associations with other nutrient intakes.

Thirteen of the 14 DMPs identified in the meta-analysis had inverse directions of effect with DNAm (Table 1), that is, decreased DNAm levels with increased metabolite biomarker levels in blood. The strongest meta-analysis signals were observed for pipecolate and DMPs cg20732160 in the body of the *PFKFB4* gene (*b* = − 0.029 ± 0.004, *p* = 1.68E−11), and cg10589813 located 751 bp upstream from *CEBPB* (*b* = − 0.029 ± 0.004, *p* = 3.49E−11). Pyridoxate was inversely associated with increased DNAm in *SLC1A5* (cg02711608 and cg22304262) and *SLC7A11* (cg06690548), with the strongest signal observed for DMP cg02711608 (*b* = − 0.038 ± 0.006, *p* = 1.63E−09) and the strongest effect size observed for DMP cg06690548 (*b* = − 0.073 ± 0.013, *p* = 1.65E−08). The association between DHA and DMP cg03440556 in the *SCD* gene was the one direct (positive) association identified in our analysis and had the strongest effect size overall (*b* = 0.093 ± 0.016, *p* = 4.07E−09).

Cg03523740 (*TXLNA*) and cg27180443 (*SCARB1*) are located in the gene promoter (TSS1500) and were associated with pipecolate (Table 1). The pyridoxate-associated DMPs in *SLC1A5* (cg02711608 and cg22304262) are in the upstream (5’) shelf of the same CpG island (chr19:47290585-47291983; Additional file 1: Table S9). Relative to *SLC1A5*, cg02711608 and cg22304262 are located on the 5’UTR, body or 1^st^ exon of the gene depending on splicing.

In the individual cohort epigenome-wide analyses the three metabolite biomarkers associated with DMPs in the meta-analysis also displayed consistent direction of association, and further signals were detected albeit in smaller subsamples. In the KORA sample alone (n = 1673), pipecolate, pyridoxate and DHA were respectively associated with 9, 4 and 1 DMPs (Bonferroni-adj. *p* < 0.05; Additional file 1: Table S10). These include the three DMPs identified with pyridoxate in the meta-analysis (cg06690548, cg02711608, cg22304262), the 1 DMP associated with DHA (cg03440556), and 4 of the 10 DMPs associated with pipecolate (Additional file 1: Table S9). In the TwinsUK sample (n = 509), associations did not surpass epigenome-wide multiple testing correction (Bonferroni-adj. *p* < 0.05). However, at a more relaxed threshold (FDR = 10%) the individual cohort analyses also detected 15 DMPs for betaine in TwinsUK (lowest *p* = 4.76E−07 for cg08960352 in the body of the *DYRK2* gene; Additional file 1: Table S11), and 126 DMPs for pipecolate in the KORA sample (lowest *p* = 5.85E−15 for cg06690548; data not shown).

If meta-analysis results were not filtered for heterogeneity among samples, pipecolate and DHA associated respectively with 3 and 1 further DMPs each (Additional file 1: Table S9; Fig. 3). Two of the high-heterogeneity DMPs associated with pipecolate included cg02711608 and cg06690548 identified for pyridoxate in the main results of our meta-analysis (Table 1).

### B vitamin intake epigenome-wide meta-analysis

Pipecolate, pyridoxate and DHA were identified as potential metabolomic biomarkers of folate and vitamin B6 (Fig. 2) and showed evidence for association with 14 DNAm signals (Fig. 3). As a follow-up validation analysis, these 14 DMPs identified in our main analysis were also tested for association with diet FFQ-derived intakes of folate and vitamin B6 in the TwinsUK, KORA FF4 and LLS cohorts (n = 2294; Additional file 1: Table S12). Of the 14 DMPs, only cg10589813, upstream the *CEBPB* gene and associated with pipecolate, reached borderline nominal significance with habitual vitamin B6 intake (*b* = − 0.023 ± 0.013, *p* = 0.06). The directions of association, while not always consistent across cohorts, were often overall consistent with the results from the biomarker EWAS meta-analysis (Additional file 1: Table S13). Of the 10 DMPs associated with pipecolate, 6 and 7 had respective consistent inverse directions of effect with habitual vitamin B6 and folate. Of the 3 DMPs associated with pyridoxate, 2 and 1 had inverse directions of effect with B6 and folate. The main DMP result coming from DHA had a consistent positive direction of effect with dietary B6. Although the metabolomic biomarkers with DMPs were associated only with folate and vitamin B6 (Figs. 1 and 2), we tested if any of the 14 DMPs were significantly associated with vitamin B12 intake as well. Only cg20732160, located in the *PFKFB4* gene and previously associated with pipecolate (a marker of folate and vitamin B6), was borderline significant with B12 (*b* = − 0.004 ± 0.002, *p* = 0.06). Nine of the 14 DMPs under study had consistent directions of effect between B12 intake and the three metabolites under study despite the metabolites marking only folate and vitamin B6 (Fig. 1).

Furthermore, an epigenome-wide association meta-analysis of FFQ-derived B vitamin intakes was performed across altogether 393,223 autosomal probes in the TwinsUK, KORA FF4 and LLS cohorts. No significant results were identified for vitamin B6 (lowest *p* = 2.68E−06) and folate (lowest* p* = 5.13E−06) and only 1 borderline significant result was found for vitamin B12: cg03473640 in the body of the *MYO5A* gene (*b* = − 0.013 ± 0.002, *p* = 1.30E−07), after multiple testing correction (Bonferroni-adj. *p* = 0.051).

### Associated gene expression results

Using previously-published results from the BIOS consortium [18] we explored whether there were expression quantitative trait methylation signals among the 14 DMPs (Table 1). Overall, methylation levels at three DMPs—cg11800635, cg12054453 and cg02711608—were associated with the expression of genes annotated to them (Additional file 1: Table S14). Methylation levels at cg11800635 and cg12054453 were inversely associated with the expression of *LOXL3/DOK1/M1AP* and *VMP1* genes, and methylation at cg02711608 was directly associated with the expression of *SLC1A5* in a BIOS subsample of 2101 individuals.

The 14 DMPs identified in this study were in or within 10kb of 23 genes, of which 15 genes had whole blood gene expression data in a sample from the TwinsUK cohort (n = 297; mean age = 63.59 ± 7.59 and mean BMI = 25.96 ± 4.63 kg/m^2^). These included *SLC1A5* and *SLC7A11* genes (associated with pyridoxate in EWAS), *SCD* gene (associated with DHA in EWAS), and 12 genes with DMPs for pipecolate. Using the 15 candidate genes identified, we explored the association between gene expression and metabolomic biomarker levels. We observed one nominally significant association between *TXLNA* expression and pipecolate in blood (*b* = − 0.136 ± 0.108, *p* = 0.015; Additional file 1: Table S15), but no signals surpassed multiple testing correction.

## Discussion

Our study identified 18 blood metabolite biomarkers of habitual folate and vitamins B6 and B12 intakes. Of these, three metabolomic biomarkers of folate and vitamin B6 showed a blood based epigenetic signature including signals in amino acid transporter genes *SLC1A5* and *SLC7A11,* and in the stearoyl-CoA desaturase gene *SCD.* These signals may give insights into mechanisms involved in B vitamin uptake and regulation within the one-carbon metabolism pathway.

The B vitamins pyridoxine (B6), folate (B9) and cobalamin (B12) are essential soluble micronutrients that influence metabolism, physiology, immunity and development in living organisms through their roles in the one-carbon metabolism pathway—a biochemical network, which produces methyl groups for DNA synthesis and methylation. B6 and B12 function as enzymatic cofactors that facilitate reactions in the folate and methionine cycles in one-carbon metabolism; folate feeds into one-carbon metabolism as the principal substrate in the folate cycle. The conversion of hcy to methionine is particularly important as circulating hcy levels have been linked to several conditions, specifically, cardiovascular disease, diabetes, cancer and cognitive function. The B vitamins are proposed to have protective effects on human health through their influences on DNAm and levels of circulating hcy [19–22].

In this study we aimed to identify metabolomic biomarkers of folate and vitamins B6 and B12 to explore in downstream epigenome-wide association analysis towards identifying DNAm signatures of B vitamin intakes. Eighteen metabolites were identified as potential biomarkers of folate and vitamins B6 and B12, with one of the profiled metabolites—pyridoxate—acting within the vitamin B6 metabolic pathway. Sensitivity analyses showed that metabolite associations were non-specific. The non-specificity was expected since foods are composed of different nutrients and there will be a correlation of intakes according to an individual’s dietary choices. However, diet quality and total energy intake were not major confounders of our analysis. In line with our results, Posma et al*.* (2020) also identified associations between intakes of B vitamins with levels of betaines and fatty acids in urine [16]. Posma et al*.* identified direct correlations between folate, B6 and proline betaine/4-hydroxyproline betaine, and inverse correlations between folate, B6 and C5-C10 fatty acids in general [16]. Here, we identified direct correlations between folate and betaine, but the fatty acids identified had distinct directions of effect in blood depending on the molecule under study.

Metabolomic biomarker findings for intakes of folate and vitamin B12 were confirmed against their corresponding circulating levels in plasma, where direction of effect matched results based on self-assessed habitual dietary data and nominal significance was achieved for 4/6 metabolomic biomarkers. Hcy and hcy-s levels were both nominally associated with 8/18 metabolomic biomarkers identified for folate, and vitamins B6 and B12. Biomarkers with positive direction of association with folate and vitamins B6 and B12 had negative directions of association with circulating hcy levels in blood, and vice-versa. This matches current knowledge that plasma concentrations of hcy are inversely related to the intake of folate, B6 and B12, and nongenetic determinants of hcy concentrations in blood include inadequate concentrations of B vitamins [5, 6, 19–22].

Using epigenetic and metabolomic data from the TwinsUK and KORA F4 cohorts we were able to meta-analyse epigenome-wide associations for 7/18 metabolite biomarkers identified. Pipecolate (a marker of folate and vitamin B6), pyridoxate (a marker of folate and vitamin B6) and DHA (a marker of vitamin B6) were respectively associated with 10, 3 and 1 DMPs.

Of the 3 blood metabolomic biomarkers identified with DMPs epigenome-wide, pyridoxate has the most immediate link to B vitamins. Pyridoxate, or 4-pyridoxic acid, is the main catabolic product of vitamin B6 metabolism, and is formed from pyridoxal in the liver [23]. Pyridoxate is excreted into urine and its concentration in plasma is directly correlated with vitamin B6 intake [24]. Its use as a biomarker of vitamin B6 had mixed results in previous studies, however, and other forms of vitamin B6 have been encouraged in clinic [24]. In this study, we observed a strong positive correlation between the intake of dietary vitamin B6 and pyridoxate measured using Metabolon Inc. Pyridoxate was the only metabolite of the subpathway of vitamin B6 metabolism in our Metabolon panel of 756 metabolites, and therefore we suggest its use as a potential biomarker of vitamin B6 intake.

DHA is an essential omega-3 fatty acid from diet that needs phosphatidylcholine for circulation in the plasma and distribution to peripheral tissues [25]. As a consequence, it takes part in one-carbon metabolism, where methyl groups are transferred from SAM during the conversion of phosphatidylethanolamine-DHA to phosphatidylcholine-DHA [25]. Folate and vitamins B6 and B12 concentrations in plasma have been previously associated with DHA in blood in a cohort of European adolescents, likely due to their role in the maintenance of the levels of SAM [26]. DHA status has itself also been associated with B vitamin supplementation, where individuals with higher levels of DHA in plasma could gain more from supplementing their diet with vitamin B12 and folic acid in order to lower their hcy levels, which are associated with aging cognitive decline [27]. Pipecolate, or pipecolic acid, is a metabolite of lysine degradation in human physiological fluids, including the blood, urine and brain, with plasma pipecolate originating from both the bacterial catabolism of dietary lysine in the intestine and the direct dietary intake of plants with high levels of pipecolic acid [28, 29]. Pipecolate levels have been associated with pyridoxine-dependent epilepsy, but direct association of B6 deficiency and pipecolic acid metabolism is unlikely [30]. Indeed, we observed a positive correlation between B6 intake and pipecolate measured in plasma in our study.

The directions of effect for the DMPs identified from pyridoxate, pipecolate and DHA were often consistent with results obtained directly from FFQ-derived B vitamin intakes in the TwinsUK, KORA FF4 and LLS cohorts. Mandaviya et al*.* [8] identified associations between dietary folate and 6 DMPs (cg23465990, cg11832534, cg03249011, cg14398883, cg00826902, cg14145338), but these were not among those identified for pipecolate and pyridoxate in our main analysis. Dietary folate was associated with hypomethylation at single sites in Mandaviya et al*.* [8]; we observed the same trend here for pipecolate and pyridoxate. Previously Petersen et al*.* [31] reported an epigenome-wide analysis of serum metabolites in the KORA F4 cohort [31]. Petersen et al*.* [31] reported that methylation at 2 CpG sites—cg16936953 and cg12054453—was significantly negatively associated with pipecolate levels in blood. In line with this result, our meta-analysis identified DMP cg12054453 as peak signal for pipecolate. DMP cg16936953 was borderline significant (Bonferroni-adj. *p* = 0.052), but did not pass heterogeneity filters in a meta-analysis of results with TwinsUK. Overall, the predominantly inverse directions of effects identified epigenome-wide in Mandaviya et al*.* [8] for dietary folate, in Petersen et al*.* [31] for pipecolate, and in our study for pipecolate and pyridoxate suggest that population-wide differences in B vitamin intake within the normal reference values can affect one-carbon metabolism homeostasis with higher B vitamin linked to lower levels of methylation. This is particularly apparent for vitamin B6, which is a cofactor in the transsulfuration pathway that converts hcy to cysteine, and lowers the production of methionine available for DNA methylation. In our study, pipecolate and pyridoxate were markers of both vitamin B6 and folate, but had stronger associations with vitamin B6. Moreover, vitamin B6 and folate intakes were highly correlated in our data (Pearson’s *r* = 0.62 for folate and B6, while *r* = 0.04 for folate and B12, and *r* = 0.16 for vitamins B6 and B12). It is thus possible that we are primarily observing the effects of vitamin B6 in one-carbon metabolism in the inverse associations reported.

The genes annotated to the DMPs identified in our meta-analysis varied in function. Pipecolate was associated with decreased methylation in genes with important roles in cellular metabolism and homeostasis. Specifically, *PFKFB4* is crucial in regulating the concentration of the glycolytic byproduct fructose-2,6-bisphosphate, while *SCARB1* is a plasma membrane receptor for high-density lipoprotein and cholesterol trafficking between cells [32, 33]. DNAm in the *PFKFB4* gene has been previously associated with the regulation of glycolytic potential in skeletal muscle [34]. DHA was associated with increased DNAm in the *SCD* gene, which encodes the Stearoyl CoA Desaturase-1 enzyme that converts saturated fatty acids into monounsaturated fatty acids and plays a role in obesity and insulin resistance. Decreased promoter methylation of the *SCD* gene has been previously linked to obesity [35].

Pyridoxate was associated with hypomethylation in amino acid transporter genes *SLC1A5* and *SLC7A11*. *SLC7A11* encodes a cysteine/glutamate antiporter system, a critical modulator of intracellular redox balance that mediates the exchange of intracellular glutamate for extracellular cystine, an essential precursor for glutathione synthesis [36, 37]. Vitamin B6-dependent enzymes also catalyse most reactions of the transsulfuration pathway, which drives homocysteine to cysteine and further into glutathione peroxidase proteins [38]. In our study pipecolate (direct marker of B6) was associated with hypomethylation in cg06690548, suggesting that vitamin B6-dependent hypomethylation in *SLC7A11* may be related to processes implicated in cysteine homeostasis and oxidative stress. Hypermethylation of cg06690548 has also recently been associated with downregulation of *SLC7A11* in Parkinson’s disease [39].

SLC1A5 is a sodium-dependent amino acid transporter with broad substrate specificity and preference for glutamine [40]. Consequently, *SLC1A5* is expressed in highly proliferative cells such as inflammatory, stem and cancer cells to meet their augmented glutamine demand. Differential methylation of cg02711608 (located in the 5’UTR region of *SLC1A5*) has been linked to alcohol consumption and BMI [41–43]. Hypomethylation of cg02711608 and cg22304262 (also in the 5’UTR region of *SLC1A5*) has been linked to higher blood pressure [44]. A putative causal effect has further been demonstrated for DMP cg22304262 in the context of incident coronary heart disease [45], as recently reviewed by us [46]. Folate intake and supplementation have been associated with improved endothelial function [47], lower systolic and diastolic blood pressure [47, 48], and overall lower risk of incident hypertension [49]. DNAm could thus fulfil a mechanistic role in the mediation of B vitamin intake and determinants of cardiovascular risk. Pyridoxate was a stronger marker of B6 than folate in our results (Fig. 2, Additional file 1: Table S2). This could partially explain why pyridoxate-associated hypomethylation of cg02711608 and cg22304262 (Table 1)—linked to high blood pressure [44]—was found in the context of this study. Cg22304262 was hypermethylated with the intake of folate measured directly from FFQs (Additional file 1: Table S13), but diet cohort results were heterogeneous and lacked the consistency of the metabolomic results.

The functional relevance of our main results was explored in the BIOS consortium and in a subsample from TwinsUK to explore methylation-expression and metabolomic-expression associations. Overall, methylation levels at cg11800635 (associated with pipecolate in EWAS), cg12054453 (associated with pipecolate in EWAS) and cg02711608 (associated with pyridoxate in EWAS) were associated with the expression levels of genes in the BIOS consortium. Furthermore, DMP cg03523740 for pipecolate is located in the promoter region of the *TXLNA* gene and in TwinsUK *TXLNA* expression changed nominally with pipecolate levels.

As metabolomic platforms become more ubiquitously used in cohort studies, we aimed to identify metabolomic biomarkers of B vitamin intake in order to circumvent limitations of accuracy associated with habitual diet measurement. Moreover, using habitual dietary data to identify B vitamin-associated metabolites resulted in larger sample sizes and more power, in comparison to using folate and B12 data measured directly from plasma in TwinsUK (n > 1000 for habitual diet and n < 730 for folate and B12 in plasma).

Both the habitual diet and blood levels of B vitamins used in this study are within the normal ranges expected for humans. As such, in future a stratified analysis of the levels of B vitamins could reveal additional metabolic and epigenetic signatures of interest. Additionally, the discovery phase of our study included only UK females and the results may not reflect biomarkers in males or in individuals of non-European ancestry. Another limitation of our study was the small overlap between the blood metabolomic data available in TwinsUK and KORA F4. We were only able to meta-analyse epigenome-wide results for 7 of the 18 blood metabolomic biomarkers initially identified. It remains unknown whether, in addition to pipecolate, pyridoxate and DHA, other B vitamin metabolomic biomarkers identified in the discovery phase of our study have epigenome-wide effects in the DNA methylome. The B vitamins intake metabolomic biomarker identified with most confidence in our study was pyridoxate, because pyridoxate is the end product of vitamin B6 metabolism before excretion from the body. However, overall the non-specificity of the metabolomic biomarkers identified, while expected due to the high correlation of nutrients in food, also limits their application in nutritional assessment.

Mandaviya et al*.* [8] reported 6 DMPs from a stratified analysis of folate intake. Unlike Mandaviya et al*.* we were unable to identify DMPs for dietary folate after correcting for multiple testing. This was probably due to differences in our approach and much lower number of samples in the EWASs of our habitual diet meta-analysis (n = 2294) compared to Mandaviya et al*.* [8] (n = 5841). Instead, our findings identified 14 epigenome-wide signals for metabolomic biomarkers of B vitamins in a more modest sample size (n = 2182), suggesting that blood metabolites may offer not only an unbiased, but also more powerful approach over self-assessed reports of dietary intakes.

## Conclusion

Using metabolomics and self-assessed dietary data we were able to identify blood metabolomic biomarkers of B vitamins with epigenome-wide association effects in whole blood DNAm. Pyridoxate—a catabolic product of the vitamin B6 metabolism—stands out as a potential blood metabolomic biomarker of B6 with noticeable epigenome-wide effects on DNAm. Significant epigenome-wide associations were observed from metabolomics data that were not observed with a similar sample size directly from self-reported dietary data. Metabolomic biomarkers of B vitamins are exact tools that can unveil novel differentially methylated signals of dietary intakes in the human epigenome.

## Methods

### Cohort information

***TwinsUK.*** The TwinsUK registry is ongoing since 1992 and includes over 15,000 research volunteer twin participants from the United Kingdom [50]. Volunteers are monozygotic and dizygotic same-sex twins, predominately female (82%), middle-aged (mean age of 59 years) and over 18 years-old. Volunteers were recruited without selecting for disease and are mostly of European descent. Information on participants has been obtained through numerous questionnaire responses and comprehensive phenotyping over the years, with the particular application of several 'omic' technologies for a range of sample types. In this study we used epigenetic, transcriptomic and metabolomic profiling in TwinsUK, together with questionnaire level data from the twins.

***KORA.*** The KORA (Cooperative Health Research in the Region of Augsburg) study is an ongoing registry of Southern German citizens with baseline recruiting dating back to 1999 (KORA S4). Selection of citizens was random with equal strata by sex and age and included 4261 subjects aged 25–74 years. Of these, KORA F4 (2006–2008) and KORA FF4 (2013/14), respectively the first and second follow-up to the S4 baseline, carried out with 3080 and 2279 participants each [51, 52]. In this study participants from the F4 follow-up were selected to explore methylation signal changes by metabolomic biomarkers, and participants from the FF4 follow-up were selected to explore methylation signal changes in response to diet, according to availability of data.

***LLS.*** The Leiden Longevity Study (LLS) is a multigenerational study that recruited nonagenarian siblings of European descent and their offspring. Altogether 944 long-lived proband siblings (mean age of 94 years), 1671 offspring (mean age of 60 years) and 744 controls (the offspring spouses, mean age of 60 years) were recruited at baseline (between 2002 and 2006). Members of long-lived families are very similar to control groups with whom they likely share similar environment, lifestyle, and age, but have more favourable morbidity and mortality outcomes [53]. Members of long-lived families were analysed as one cohort of middle-aged people and the current study was restricted to unrelated individuals in epigenetic analyses.

### Data collection and processing

#### Habitual B vitamin intakes

The habitual intakes of folate and vitamins B6 and B12 of participants was measured using food frequency questionnaires (FFQs) in the TwinsUK and LLS cohorts, and a blended approach comprising repeated 24h food lists and an FFQ in the KORA FF4 cohort.

***TwinsUK.*** Food frequency questionnaires used in the TwinsUK study comprised 131 food and drink items from the EPIC Norfolk study [54]. Processing of these data has previously been described [55], and data were available for 3157 female twins. The daily intake of each item was calculated in g/day using the FETA software [56] and the default nutritional database based on the McCance and Widdowson’s The Composition of Foods (5th edition) [57]. The residual method was used to obtain B vitamin intake estimates independent of total energy intake [58]. In addition to B vitamins, the daily intakes of 38 other nutrients was estimated for use in sensitivity analysis of B vitamin intake associations. The 38 other nutrients quantified included altogether 16 macronutrients (i.e. total protein, total fat, total carbohydrates, starch, total sugars, glucose, fructose, sucrose, maltose, lactose, non‐starch polysaccharides, saturated fats, monounsaturated fats, polyunsaturated fats, trans fats and cholesterol), 11 minerals (i.e. sodium, potassium, calcium, magnesium, phosphorus, iron, copper, zinc, chloride, manganese and iodine), and 11 other vitamins/vitamin nutrient precursors (i.e. retinol, carotene, vitamin C, vitamin D, vitamin E, thiamine, riboflavin, niacin, tryptophan, pantothenate and biotin). The overall diet quality of the TwinsUK participants was calculated using the Alternate Healthy Eating Index 2010 (AHEI-2010) diet score [17], which ranges 0–10 and scores positively the intake of healthy foods (e.g., whole grains and healthy fats) and scores negatively the intake of unhealthy foods (e.g., red and processed meats). The AHEI-2010 accounts for the participants alcohol intake and was calculated here for the sensitivity of overall diet quality.

***KORA FF4.*** Repeated 246-item 24-h food lists derived from the NAKO Health study [59] and 148-item FFQs adapted from the German version of the multilingual European Food Propensity Questionnaire [60] were used in the KORA FF4 study. The processing of these data was first described elsewhere [61], and data was available for 1602 participants. Classification of dietary intakes in KORA was performed with the EPIC-Soft software [62] and B vitamin intake data was calculated based on the German food composition database Bundeslebensmittelschlüssel, version 3.01 [63]. Like in TwinsUK, the residual method was used to get B vitamin intake estimates independent of energy intake in KORA FF4.

***LLS.*** Food frequency questionnaires used in the LLS study included 218 items constructed from the 104-item VetExpress FFQ combined with the Dutch National Food Survey [64]. B vitamin intake data was estimated in grams per day using the NEVO table 2011 [65] as reference panel. A weighted average was calculated for the nutrient composition of a food item, based on the consumption of each NEVO product included in the food item according to the Dutch National Food Consumption Survey 2010. Dietary intake data in grams per day was collected from 1716 individuals.

The energy-adjusted intakes of folate and vitamins B6 and B12 were used in the discovery phase of our study to identify metabolomic biomarkers of B vitamins in participants from TwinsUK. Energy-adjusted intakes from TwinsUK, LLS and KORA FF4 were used for the epigenome-wide association meta-analysis of habitual B vitamin intakes. B vitamin outlier values were removed across analyses in similar fashion, where outliers 3 standard deviations away from the mean were excluded from the subsamples.

#### Blood levels of folate, vitamin B12 and homocysteine

Measured blood levels of folate (ng/mL) and B12 (ng/L) were available in the TwinsUK cohort for a subset of participants with metabolomics data. Homocysteine levels (µmol/L) were also available in plasma and serum. Overall, and after removing outliers 3 standard deviations away from the mean, a total of 729, 718, 473, and 707 individuals had circulating folate, vitamin B12, hcy and hcy-s levels measured within 2 years of metabolomics profiling, respectively.

#### Whole blood metabolome

Blood metabolites used in this study were profiled in the TwinsUK and KORA F4 cohorts using the Metabolon platform. Metabolon is a chromatography mass spectrometry platform that produces semiquantitative data where standards are used to determine the retention time and relative intensity of metabolites.

***TwinsUK.*** Fasting blood serum samples were collected from female participants and profiled using the Metabolon platform (Metabolon, Inc., Durham, NC). The processing of samples has previously been described [66]. Metabolomic data were median-normalised by dividing metabolite concentrations by the day median of that metabolite and then rank inverse-normalised. Metabolites with more than 20% of missing values were excluded and minimum run day measures were imputed to the missing values. A total of 756 metabolites were kept for analysis from a total of 6196 samples taken from 2069 female twins spanning several years. Of the 756 metabolites, 591 (78%) are annotated and fall into the broad metabolic groups of amino acids, carbohydrates, cofactors and vitamins, energy, lipid, nucleotide, peptide, and xenobiotics. One of the profiled metabolites, pyridoxate, is known to act within the vitamin B6 metabolic pathway. A subset of 1063 (for folate and vitamin B6) and 1064 (for vitamin B12) female twins had a blood metabolomic profile within 2 years of FFQ. These twins were used for biomarker discovery in the TwinsUK sample.

***KORA F4.*** Fasting blood serum samples were collected from participants of the KORA F4 (Cooperative Health Research in the Region of Augsburg) study population and profiled using the Metabolon platform (Metabolon, Inc., Durham, NC). The processing of samples was previously described [67, 68]. Like in TwinsUK, metabolomics data in KORA F4 was median-normalised by dividing metabolite concentrations by the day median due to fluctuations in the data caused by instrument maintenances that are day-dependent. Then in KORA each metabolite data was multiplied with their overall median values and log transformed. To match the TwinsUK outcome variables and for the purpose of meta-analysis, KORA data was normalised by rank-based inverse normal transformation in this study. Overall, and after quality control, 276 metabolites in human serum were profiled from 1768 participants of the KORA F4 population.

#### Whole blood DNA methylation

***TwinsUK.*** Fasting whole blood DNAm of 990 individuals was profiled using the Infinium HumanMethylation450 BeadChip (Illumina Inc, San Diego, CA). DNAm was assessed at > 450,000 sites and processing of methylation signals was performed with R Bioconductor software [69]. Briefly, the ENmix package [70] was used for quality control of the data, and the *minfi* package [71] was used to exclude samples with median methylated and unmethylated signal ratio < 10.5. Background correction, dye bias correction and quantile normalization were performed with ENmix as previously described [72]. Underperforming probes and outlier samples were identified using standard parameter values and signals with detP > 0.000001 and nbead < 3 were excluded from the analysis. Maximum probe and sample missingness were set to 5%. Methylation beta-values (ranging 0–1 for un- to fully-methylated) were estimated with ENmix while adjusting for array probe type bias with the Regression on Correlated Probes (RCP) method [73]. Methylation beta-values were converted to methylation M-values with the *lumi* package [74] prior to downstream analysis for better statistical validity of the models. A total of 487 and 509 females had DNAm measures within 2 years of FFQ and 5 years of metabolomic profiling, respectively. The two subsamples were used in downstream analyses.

***KORA.*** Fasting whole blood DNAm was available in the KORA F4 and FF4 waves used in this study for metabolomic and habitual diet intake analysis, respectively. ***KORA F4.*** Whole blood DNAm was measured with the HumanMethylation450 BeadChip and processing of data was previously described [75]. Briefly, the methylation data was extracted through Illumina’s Genome Studio (version 2011.1) methylation module (v1.9.0) and processed with the CPACOR pipeline [76]. Background correction was performed with *minfi* [71] and bad signals were excluded if detP > 0.01. Maximum sample missingness was set to 5% and methylation beta-values were estimated after quantile normalisation of the data. ***KORA FF4.*** Whole blood DNAm was measured with the Infinium MethylationEPIC BeadChip, which assesses methylation at > 850,000 sites of the human genome. Quality control of this data was previously described [77] and processed in similar fashion to DNAm in the KORA F4 population (i.e. following the CPACOR pipeline). KORA F4 and FF4 methylation data was converted to M-values prior to analysis in this study. A total of 1673 and 1322 participants respectively of KORA F4 and FF4 had a metabolomic profile and FFQ collected in the same wave as whole blood DNAm and were used in downstream analysis.

***LLS.*** Fasting whole blood DNAm was available for 732 individuals of the LLS cohort. Processing and normalization of the data were done as described in the DNAmArray workflow (https://molepi.github.io/DNAmArray_workflow/). Briefly, methylation data was extracted using the *minfi* package [71] and sample-level quality control was performed using MethylAid [78]. Signal exclusion was performed based on detP > 0.01, nbead < 3 and zero values for intensity. Functional normalization of the data was performed using five principal components extracted using the control probes. Maximum sample missingness was set to 5% and methylation beta-values were converted to M-values to match other cohorts in this study. A total of 485 long-lived participants of the LLS study had DNAm and FFQ and were used in this study for the habitual B vitamin intake epigenetic meta-analysis.

Across cohorts, only autosomal probes were kept for analysis in this study. Polymorphic or probes that mapped to multiple locations in the genome were also removed. Altogether a total of 430,768 and 393,223 probes were identified in TwinsUK/KORA F4 and TwinsUK/KORA FF4/LLS cohort groups, respectively, and kept for the biomarker and habitual diet epigenetic meta-analyses.

#### Whole blood gene expression

Gene expression data used in this study was profiled in the TwinsUK cohort. Fasting whole blood transcriptomic data was obtained using Illumina RNA-Seq technologies (Illumina, Inc., San Diego, CA). There data and processing have previously been described [79]. Briefly, the STAR software v2.4.0.1 [80] was used to align reads to the hg19 reference genome and only uniquely mapped properly paired reads were kept after alignment. GENCODE annotation v19 gene counts were obtained with featurecounts [81], and then standardised with trimmed mean of M-values (TMM)-adjusted counts per million (CPMs) and inverse-normalised prior to downstream analysis. Only genes with at least 0.5 CPM expressed in 90% of samples were kept in the original data. A total of 23 genes were manually annotated to the 14 DMPs identified using the UCSC genome browser (hg19) selecting for genes ± 10 kb away from the CpG site. Fifteen out of the 23 genes from our main analysis were present in the data. A total of 297 female twins had gene expression data profiled within 5 years of metabolomic profiling. The 15 genes and 297 female twins identified were used for follow-up gene expression analysis in the TwinsUK cohort.

### Statistical analyses

#### Discovery phase

Metabolomic data collected within 2 years of FFQ were used for the discovery of B vitamin metabolite biomarkers in the TwinsUK cohort. A total of 1063 (for folate and vitamin B6) and 1064 (for vitamin B12) female twins were included in this analysis after removing outliers. Twins were either monozygotic or dizygotic, and zygosity was included as a factor in the model to account for the level of shared genetic variation (i.e. MZ share approximately 100% while DZ share 50% of genetic variation). Twins with their co-twin in a twin pair missing were reclassified as unrelated individuals. Metabolome-wide associations between folate and vitamins B6 and B12 were separately undertaken for the 756 metabolites from Metabolon. Linear regression mixed-effects models were applied using the lme4 package [82]. Models were adjusted for the participants’ age and BMI, the time interval between food questionnaire and metabolomic sample collection, and the family and zygosity of participants as random effects. In this instance the energy-adjusted intake of a B vitamin was the predictor and the inverse-normalised signal of a metabolite was the outcome. Slight variations in final sample sizes were due to missing metabolomic data although each metabolite was profiled in n > 1000 in most cases (n < 1000 for 3 metabolites; lowest n = 976). Multiple testing adjustment of each B vitamin result was applied using Bonferroni correction (*p* = 0.05/756 tests = 6.61E−05, for Bonferroni-adj. *p* < 0.05). Structurally unidentified metabolites (unknowns) were discarded. Metabolites with single asterisk were annotated based on in silico prediction. A total of 18 metabolites were kept for downstream analyses.

#### Sensitivity analyses

Three sensitivity analyses were performed on the 18 putative biomarker metabolites identified during the biomarker discovery phase.

***Total energy intake and diet quality.*** To assess their putative impact on the identification of biomarker metabolites, the biomarker discovery model described immediately above was extended to further include the total energy intake and overall diet quality of the participants as covariates. Here, the AHEI-2010 diet score [17]and total energy consumed (in kcal/day) were included as fixed effect variables. Multiple testing significance was presented with Bonferroni correction (*p* = 0.05/756 tests, Bonferroni-adj. *p* < 0.05).

***Other nutrients.*** To determine the specificity of our findings, a panel of 38 other nutrients common from habitual diet were used in associations with the 18 biomarker metabolites identified in our main analysis. In this instance the other nutrient (e.g. glucose, iron, vitamin C, etc.) replaced the predictor variable in the original model, and the metabolite biomarker remained as the outcome. Predictor outliers were removed as previously described (n > 1000 in all instances). Only associations for the 18 metabolite biomarkers were performed per nutrient, but Bonferroni thresholds used to determine the significance of these results were set metabolome-wide as previously described (i.e. for each nutrient analysis adj. *p* = 0.05/756 tests).

***Blood chemistry.*** To evaluate if B vitamin findings from habitual diet could be validated using the plasma or serum levels of B vitamins and homocysteine, associations were performed in subsamples of 473–729 twins with available folate, vitamin B12, hcy and hcy-s data. Circulating folate, vitamin B12 and homocysteine levels were used as predictors and associations were performed for the metabolites identified as the outcome. The 5 and 1 metabolites associated with dietary folate and vitamin B12 were used here in associations with folate and B12 levels in plasma, respectively. All 18 metabolites identified overall in our main analysis were used for the hcy and hcy-s associations. Linear mixed effects models were adjusted for age, BMI, time interval between blood metabolomics and other blood chemistry data, and family and zygosity of the twin sample. The significance of each metabolite result was determined with the Bonferroni correction threshold extrapolated previously from the full 756 metabolite panel (*p* = 0.05/756 tests).

#### Epigenome-wide association meta-analyses

***B vitamin metabolite biomarkers.*** An epigenome-wide association study (EWAS) was performed in the TwinsUK cohort for each of the 18 metabolite biomarkers identified in the discovery phase of our study. Seven of the 18 metabolites identified in TwinsUK (i.e. 1-docosahexaenoyl-GPC (22:6)*, 7-methylguanine, betaine, pipecolate, pyridoxate, uridine and DHA) were also represented in the KORA F4 Metabolon data and EWASs results were meta-analysed between TwinsUK (n = 509) and KORA (n = 1673; total n = 2182). Epigenome-wide association studies were performed using linear regression mixed effects models where DNAm M-values were the outcome and inverse-normalised metabolite levels in blood were predictors. Metabolite levels 3 standard deviations away from the mean were excluded and models were adjusted for age, BMI, trichotomous smoking (0: never smoker; 1: ex-smoker; 2: current smoker), blood cell proportions (lymphocytes, granulocytes and monocytes), time interval between blood metabolomics and methylation profiling, and technical and cohort-specific variables such as family and zygosity in TwinsUK, and sex (0: female; 1: male) in the KORA F4 cohort. In TwinsUK, blood cell proportions were estimated with the Houseman method [83] using Horvath’s DNA Methylation Age Calculator [84], with lymphocytes corresponding to the sum of CD8T, CD4T, NK and B cell type proportions. Associations were performed across 430,768 autosomal probes and results were meta-analysed with METAL [85]. Here, the effect sizes and standard errors obtained in TwinsUK and KORA were used to conduct a fixed-effects inverse variance weighted meta-analysis. The heterogeneity of results was analysed with METAL across the two cohorts. The significance of results was established based on the Bonferroni correction (*p* = 0.05/430,768 tests = 1.17E−07, for Bonferroni-adj. *p* < 0.05) and sample heterogeneity (HetISq < 75% and HetPval ≥ 0.05). The false discovery rate method was used to explore results further in each cohort with *q* < 0.1. Genes of DMPs identified were manually annotated using the UCSC genome browser (hg19) selecting for genes ± 10 kb away from the CpG site.

***B vitamin intakes.*** An EWAS of the habitual intake of folate and vitamins B6 and B12 was performed in the TwinsUK (n = 487), KORA FF4 (n = 1322) and LLS (n = 485) cohorts and meta-analysed in this study (total n = 2294). Analyses were performed using methylation M-values as the outcome variable and the energy-adjusted habitual diet intake of B vitamins as the predictor. B vitamin intakes 3 standard deviations away from the mean were excluded prior to analysis and models were adjusted for age, BMI, trichotomous smoking, blood cell proportions, and technical and cohort-specific variables. Meta-analysis of results was performed with METAL as described above (*p* = 0/393,223 tests = 1.27E−07, Bonferroni-adj. *p* < 0.05). Pipecolate, pyridoxate and DHA were identified as metabolomic biomarkers of folate and vitamin B6, and DMPs associated with these compounds were explored in further detail in context of habitual diet.

### Gene expression follow-up

Methylation-expression associations for the DMPs identified in this study were explored in previously-published data from the BIOS (Biobank-Based Integrative Omics Studies) consortium in The Netherlands [18]. *Cis* expression quantitative trait methylation signals captured at FDR = 5% across 2101 samples were extracted from the “2015_09_02_cis_eQTMsFDR0.05-CpGLevel.txt” file hosted in the BIOS QTL browser (https://molgenis26.gcc.rug.nl/downloads/biosqtlbrowser/).

A targeted metabolomic-gene expression follow-up analysis of 15 genes with DMPs was performed in the TwinsUK cohort (n = 297). Levels of *PFKFB4, CEBPB, DOK1, LOXL3, M1AP, LOC646329, MIR29B1, TXLNA, KPNA6, VMP1, DYRK2* and *SCARB1* expression were tested for association with levels of pipecolate. Levels of *SLC1A5* and *SLC7A11* expression were tested for association with pyridoxate, and *SCD* expression levels were tested in association to DHA. Linear regression mixed effect models were implemented where inverse-normalised metabolite levels were included as the predictor and the inverse-normalised TMM-adjusted gene counts were included as the outcome. Models were adjusted for age, BMI, trichotomous smoking, time interval between blood metabolomics and gene expression profiling, and other technical covariates (fixed: insert-size median and mean GC content; random: primer index, date of sequencing and RNA extraction batch) used previously [86]. Family and zygosity were included as random effects. Multiple testing correction was applied as previously described.

### Supplementary Information

**Additional file 1: Table S1.** Cohort characteristics for the B vitamin metabolomic biomarker discovery.** Table S2**. Blood metabolites associated with the dietary intake of folate (B9) and vitamins B6 and B12 (metabolome-wide Bonferroni-adj.* p* < 0.05). **Table S3**. B vitamin metabolomic biomarker associations adjusted for diet quality (AHEI-2010) and total energy intake (metabolome-wide Bonferroni-adj.* p* < 0.05).** Table S4**. B vitamin metabolomic biomarker associations with other nutrient intakes (metabolome-wide Bonferroni-adj.* p* < 0.05).** Table S5**. Summary of nutrients associated with the B vitamin metabolomic biomarkers identified (metabolome-wide Bonferroni-adj.* p* < 0.05).** Table S6**. B vitamin metabolomic biomarker associations with circulating blood levels of folate (B9), vitamin B12, hcy and hcy-s.** Table S7**. Metabolite associations with circulating blood levels of folate (B9), vitamin B12, hcy and hcy-s (metabolome-wide Bonferroni-adj.* p* < 0.05).** Table S8**. Cohort characteristics for the B vitamin metabolomic biomarker epigenome-wide meta-analysis.** Table S9**. Annotation of the epigenome-wide association meta-analysis of folate (B9) and vitamins B6 and B12 biomarker metabolites (epigenome-wide Bonferroni-adj.* p* < 0.05).** Table S10**. Epigenome-wide association of folate (B9) and vitamins B6 and B12 biomarker metabolites in the KORA F4 cohort (epigenome-wide Bonferroni-adj.* p* < 0.05).** Table S11**. Epigenome-wide association of folate (B9) and vitamins B6 and B12 biomarker metabolites in the TwinsUK cohort (epigenome-wide FDR = 10%).** Table S12**. Cohort characteristics for the B vitamin intake epigenome-wide meta-analysis.** Table S13**. DMP results in DMP results in the epigenome-wide association meta-analysis of folate (B9) and vitamin B6 dietary intakes.** Table S14**. Cis expression quantitative trait methylation signals from the BIOS consortium for the DMP identified in this study. **Table S15**. Gene expression results for the genes annotated in the biomarker metabolite EWAS meta-analysis.

**Additional file 2: Figure S1.** Correlation matrix of the serum levels of the metabolomic biomarkers identified.** Figure S2**. Pathway annotations of the 18 metabolomic biomarkers identified for folate and vitamins B6 and B12 (Bonferroni-adj.* p* < 0.05).

## Data Availability

Many of the blood data analysed in TwinsUK is available through GEO GSE62992 and GSE121633 for methylation and EGA EGAD00001001088 for gene expression. Additional TwinsUK individual-level data are not permitted to be shared or deposited due to the original consent given at the time of data collection. However, access to these data can be applied for through the TwinsUK data access committee. For information on access and how to apply http://twinsuk.ac.uk/resources-for-researchers/access-our-data/. The informed consents given by KORA study participants do not cover data posting in public databases. However, data are available upon request from KORA Project Application Self-Service Tool (https://helmholtz-muenchen.managed-otrs.com/external/). Data requests can be submitted online and are subject to approval by the KORA Board. LLS DNA methylation data are available upon request via the BIOS consortium (https://www.bbmri.nl/acquisition-use-analyze/bios). FFQ data is available upon request.

## Abbreviations

AHEI-2010: Alternate healthy eating index 2010
BMI: Body mass index
DHA: Docosahexaenoate
DMP: Differentially methylated position
DNMT: DNA methyltransferase
EWAS: Epigenome-wide association study
FDR: False discovery rate
FFQ: Food frequency questionnaire
Hcy: Homocysteine measured in plasma
Hcy-s: Homocysteine measured in serum
R^2^_c_: Conditional R squared
SAH: S-adenosylhomocysteine
SAM: S-adenosylmethionine
