# Supplementary material for: Metabolomic biomarkers of habitual B vitamin intakes unveil novel differentially methylated positions in the human epigenome
Source: Clin Epigenetics. 2023 Oct 19;15:166. doi: 10.1186/s13148-023-01578-7 (PMC10588110; doi:10.1186/s13148-023-01578-7)
Supplement: Supplementary file 2 — Additional file 2: Figure S1. Correlation matrix of the serum levels of the metabolomic biomarkers identified. Figure S2. Pathway annotations of the 18 metabolomic biomarkers identified for folate and vitamins B6 and B12 (Bonferroni-adj. p < 0.05). [file 13148_2023_1578_MOESM2_ESM.docx]

# Supplementary Figures


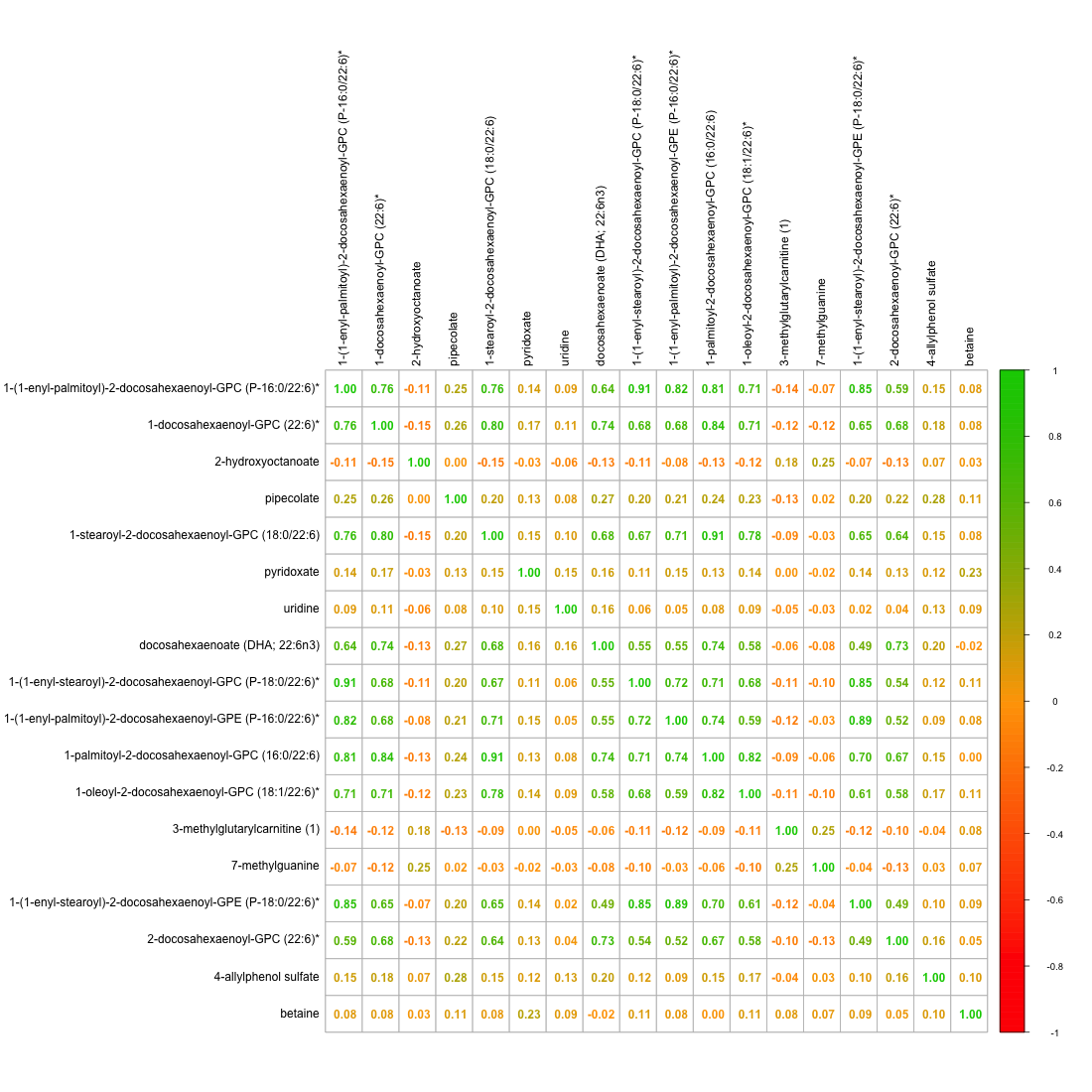


**Supplementary Figure 1.** Correlation matrix of the serum levels of the metabolomic biomarkers identified.


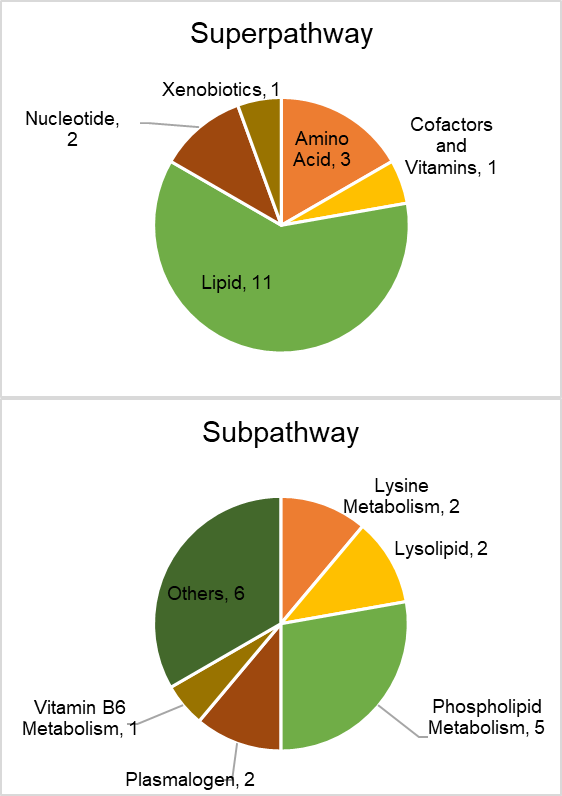


**Supplementary Figure 2.** Pathway annotations of the 18 metabolomic biomarker identified for folate and vitamins B6 and B12 (Bonferroni-adj. *p* < 0.05).
